# Bibliometric and visualization analysis of exercise interventions for respiratory infectious diseases (2000–2025): Trends, hotspots, and future directions

**DOI:** 10.1097/MD.0000000000050013

**Published:** 2026-07-31

**Authors:** Qiugang Zheng, Jindong Chen, Na Wang, Mengnan Hou, Xiurong Xu, Zan Liu

**Affiliations:** a Department of Rehabilitation Medicine, The Second Affiliated Hospital of Hainan Medical University, Haikou, Hainan, China; b The Second Clinical College, Hainan Medical University, Haikou, Hainan, China; c Department of Anesthesiology, Hainan General Hospital, Haikou, Hainan, China; d Department of Tropical Medicine, The Second Affiliated Hospital of Hainan Medical University, Haikou, Hainan, China.

**Keywords:** bibliometrics, CiteSpace, immunity, pulmonary rehabilitation, respiratory infectious diseases, telerehabilitation, visualization analysis

## Abstract

**Background:**

Respiratory infectious diseases impose a heavy burden on global health. Exercise-related interventions have attracted increasing research attention, but the knowledge landscape and emerging trends in this field have not yet been comprehensively mapped. This study aims to address this gap through a bibliometric and visualization analysis of the literature published between 2000 and 2025.

**Methods:**

A search was conducted in the Web of Science Core Collection and PubMed databases, incorporating relevant English-language literature published between January 1, 2000, and March 31, 2025. After deduplication and screening, 944 articles were ultimately included. Co-occurrence network, clustering, and emergence analyses were conducted using CiteSpace 6.4.R1 software. Descriptive statistical analyses of publication volume and country, institutional, and author distribution were performed using Microsoft Excel.

**Results:**

Publication output in this field has increased significantly since 2020, peaking in 2022, a trend highly consistent with the progression of the coronavirus disease 2019 (COVID-19) outbreak. Arena Ross, the University of London (United Kingdom), and the United States emerged as the most prolific author, institution, and country, respectively. Frequent keywords include “Physical activity,” “Pulmonary rehabilitation,” “Exercise,” “COVID-19,” “Coronavirus disease,” “Rehabilitation,” and “Quality of life.” Keywords exhibiting high burst strength include “COVID-19 (30.04),” “Coronavirus disease (10.19),” and “respiratory tract infections (7.95).” Emerging research hotspots concentrate primarily on 3 domains: “respiratory function,” “Telerehabilitation,” and “dyspnea.”

**Conclusion:**

Research on physical activity interventions for respiratory infectious diseases has shown sustained growth over the past 25 years, undergoing significant structural shifts during the COVID-19 pandemic. This study provides a broad overview of the knowledge landscape and developmental trajectory in this field.

## 1. Introduction

Respiratory infectious diseases (such as pulmonary tuberculosis, coronavirus disease 2019 [COVID-19], and pneumonia) pose a major global public health challenge owing to their high incidence rates and complex pathological sequelae. In 2019, global new cases of upper respiratory infections reached 17.2 billion (accounting for 42.83%), while 2021 saw approximately 344 million new cases of lower respiratory infections, including pneumonia, influenza, and respiratory syncytial virus. The disability-adjusted life year rate ranged from 347.67 per 100,000 in China to 1168.8 per 100,000 globally.^[1–6]^ Such diseases not only cause health impairments during the acute phase but also trigger long-term functional decline, reduced exercise tolerance, and increased risk of chronic obstructive pulmonary disease, significantly diminishing patients’ quality of life. For instance, over 70% of patients with tuberculosis (TB) experience sequelae, while approximately 80% of COVID-19 patients report persistent symptoms. Notably, approximately 50% of lower respiratory infection-related deaths can be attributed to chronic sequelae from prior infections.^[7]^ With China’s aging population, the burden of respiratory infections among individuals aged ≥60 years is projected to increase by 71.85% by 2036. The prevalence of respiratory infectious diseases is expected to increase persistently, necessitating the development of effective prevention and rehabilitation strategies.^[2]^

In recent years, exercise and physical activity have received significant research attention as non-pharmacological interventions for the management of respiratory diseases. Previous studies have reported that moderate-intensity exercise may improve respiratory function and exercise tolerance and is associated with changes in certain inflammation-related biomarkers. From a biological mechanisms perspective,^[8–12]^ regular exercise systematically induces an anti-inflammatory phenotype in the immune system through the release of myokines, such as interleukin-6, from skeletal muscle, while simultaneously optimizing immune surveillance and defense efficiency by enhancing the metabolic functions of immune cells.^[13,14]^ A wealth of research provides a solid theoretical foundation for the focus of this study. However, this evidence primarily originates from specific disease cohorts or from individual clinical and basic research studies. Its applicability across different respiratory infectious diseases and the evolution of research priorities remains lacking in a systematic, comprehensive analysis.

Visual data analysis methods based on bibliometrics can reveal the knowledge structure, research hotspots, collaborative networks, and temporal evolution trends within specific research fields at a macro level. This approach supplements the limitations of traditional narratives and systematic reviews in grasping the overall landscape.^[15–17]^ To facilitate an in-depth discussion of research findings and short-term future trends in rehabilitation exercise interventions for respiratory infectious diseases, this study employs bibliometrics and visualization analysis as its core research methodology. Using CiteSpace, Microsoft Excel, and other software tools, we systematically analyzed the research progress in this field from January 2000 to March 2025, constructing a knowledge mapping framework. The research objectives are: identify core authors, institutions, and national collaboration networks; describe the primary research hotspots and their evolutionary trajectories; and based on bibliometric findings, summarize the emerging frontier themes in recent years.

## 2. Materials and methods

### 2.1. Search methods

This study strictly adhered to the preliminary guideline for reporting bibliometric reviews of the biomedical literature checklist for reporting bibliometric reviews.^[18]^ The Web of Science Core Collection (WOSCC) and PubMed were selected as data sources (the 2 are complementary in terms of coverage and document type, with data suitable for conducting bibliometric and visual mapping analyses).^[16]^ The search was conducted on April 7, 2025, using database-specific search strategies within a publication date range of January 1, 2000, to March 31, 2025. Detailed search strategies are presented in Table 1. It should be noted that WOSCC and PubMed differ in their subject-term structures, field configurations, and retrieval logic. Consequently, this study constructed separate retrieval formulas for each database to balance retrieval sensitivity with thematic relevance. Because WOSCC and PubMed differ in indexing systems and field structures, database-tailored search strategies were applied (Table 1). The WOSCC strategy primarily used topic-field keywords to maximize sensitivity, whereas the PubMed strategy combined Medical Subject Headings terms with title/abstract terms to improve biomedical specificity. To reduce topic dilution in WOSCC, an outcome-related term block (e.g., effect, outcome, impact) was incorporated as an additional relevance filter (Table 1). Owing to the inherent delays between publication and database indexing, the literature from the first quarter of 2025 (January–March) may not be fully captured. Consequently, the 2025 publication volume should be regarded as an emerging trend rather than comprehensive annual data. Nevertheless, to enhance the timeliness of the analysis, these recent records were included while confirming that they did not alter the core trends or conclusions of the primary literature from 2000 to 2024.

**Table 1:** Search strategy.

Search settings	WOSCC search string (stepwise)	PubMed search string (final query)
#1 (exercise/rehabilitation)	TS = (exercis*) OR TS = (rehab*) OR TS=(“physical therap*”) OR TS = (physical activit*) OR TS = (training program*) OR TS = (breathing exercise*) OR TS = (kinesiotherapy) OR TS = (motor activit*) OR TS = (pulmonary rehabilitation) OR TS = (physical training) OR TS = (respiratory muscle training) OR TS = (aerobic exercise*) OR TS = (respiratory rehabilitation) OR TS = (rehabilitative training) OR TS = (lung training) OR TS = (resistance training) OR TS = (aerobic training) OR TS = (physical exercise) OR TS = (rehabilitation) OR TS = (exercise training)	Final query executed in PubMed: (((Exercise[Mesh] OR Exercise Therapy[Mesh] OR Sports[Mesh]) OR (exercis*[Title/Abstract] OR physical activit*[Title/Abstract] OR physical train*[Title/Abstract] OR rehabilitation exercise[Title/Abstract])) AND ((Respiratory Tract Infections[Mesh] OR bronchitis[Title/Abstract]))) AND (journal article[Publication Type] OR review[Publication Type]) NOT (Letter[Publication Type] OR Comment[Publication Type] OR Editorial[Publication Type] OR News[Publication Type] OR Case Reports[Publication Type])
#2 (respiratory infectious diseases)	TS = (respiratory tract infection*) OR TS = (viral respiratory infection*) OR TS = (pneumonia) OR TS = (influenza) OR TS = (respiratory infection*) OR TS = (lower respiratory infection) OR TS = (tuberculosis) OR TS = (COVID-19) OR TS = (Sars-cov-2)	(Not applicable; PubMed search was executed as a single final query shown above.)
#3 (outcome-related terms)/Final combination	#1 AND #2 AND TS = (effect* OR impact OR outcome* OR improve* OR reduce* OR exacerbate OR risk)	(Not applicable; PubMed search was executed as a single final query shown above.)
Limits/filters	Language: English; Publication years: January 1, 2000–March 31, 2025; Document types: Article OR Review	Language: English; Publication years: January 1, 2000–March 31, 2025; Publication types: journal article OR review; Exclusions: Letter/Comment/Editorial/News/Case Reports
Search date	April 7, 2025	April 7, 2025

MeSH = Medical Subject Headings, SARS-CoV-2 = severe acute respiratory syndrome coronavirus 2, TS = topic search, WOSCC = Web of Science Core Collection.

### 2.2. Inclusion and exclusion criteria

**Figure 1.**
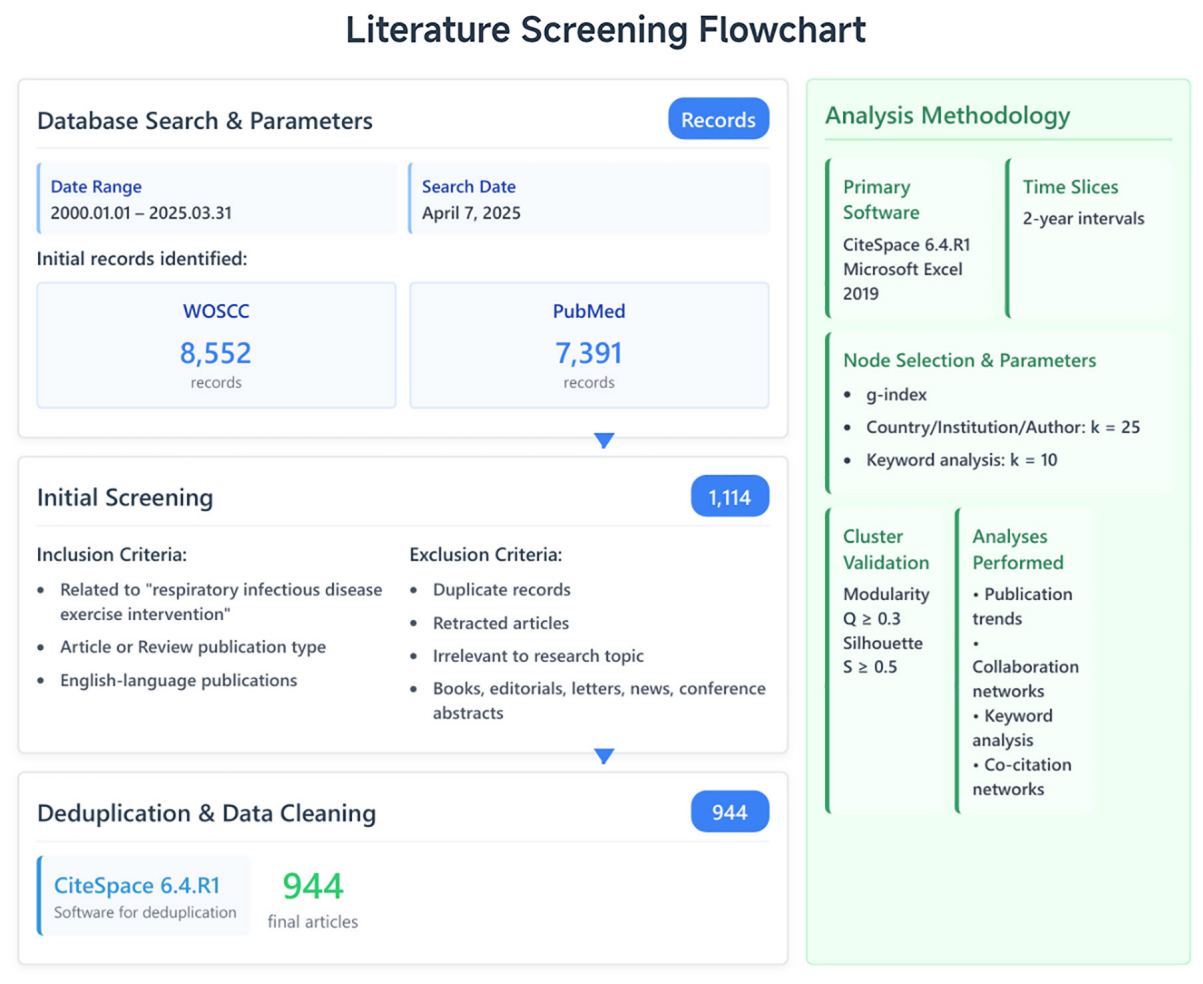
Literature screening flowchart.

All retrieved records from WOSCC (n = 8552) and PubMed (n = 7391) were screened using a multistage process (title/abstract screening followed by full-text assessment when needed). Three reviewers independently assessed eligibility, and disagreements were resolved through discussion. The complete screening process is shown in Figure 1. In addition, the literature screening was conducted independently by 3 authors, who strictly adhered to the inclusion and exclusion criteria throughout the process to ensure that the topics and research content of the studies met the inclusion criteria.

Inclusion criteria: literature related to the research topic of “exercise interventions for respiratory infectious diseases”; publication types limited to Article or Review; and English-language publications.

The exclusion criteria were as follows: duplicate publications, retracted articles, or literature irrelevant to the research topic; and publications such as books, newspapers, comments, case reports, editorials, monographs, letters, news items, and conference abstracts.

### 2.3. Research methodology

After rigorous manual screening by the research team, the 1114 articles identified by the 2 systems that met the inclusion criteria were exported in formats compatible with CiteSpace: the WOSCC database was exported in plain text format, and the PubMed database was exported in NBib format. The exported files were processed using CiteSpace 6.4.R1 to deduplicate and clean the data for the included articles from both databases (including merging duplicate records, field standardization, and identifying anomalous entries). After deduplication, a total of 944 valid articles were obtained as the final analysis sample. The complete literature screening process is shown in Figure 1. This study used CiteSpace 6.4.R1 (information visualization software developed by Professor Chao-Mei Chen of Drexel University’s College of Computing and Information) as the primary analytical tool. Microsoft Excel 2019 served as a supplementary tool for statistical and descriptive analyses, facilitating quantitative analysis and visual mapping of publication volume, countries, institutions, authors, keywords, and co-cited references.^[15,16]^

The sample for this study comprised 944 literature items screened between 2000 and 2025. Considering both the sample size and methodologies employed in prior bibliometric research, the temporal slice was set at 2 years to ensure adequate temporal resolution in the visualization while mitigating the risk of network fragmentation. Node selection was performed using the g-index to balance the representation between highly cited and emerging research nodes.

Given the varying node counts across the analyses, the keyword nodes exhibited greater diversity. Consequently, for country, institution, and author analyses, the CiteSpace default parameter (*K* = 25) was employed to ensure network structural integrity and visualization of primary collaborative relationships. For keyword analysis, given the substantial number of nodes, the g-index parameter was adjusted to *K* = 10 to reduce network noise and highlight high-frequency and core themes, thereby enhancing the focus on the major research hotspots. The validity of the clustering results was assessed using modularity *Q* and silhouette coefficient *S*, where *Q* ≥ 0.3 and *S* ≥ 0.5 were deemed indicative of acceptable distinction and internal consistency within the clustered structures.^[15,16]^ It should be noted that bibliometric and visualization outcomes are, to some extent, dependent on parameter settings and algorithmic characteristics. Consequently, this study primarily employed a descriptive approach to interpret network structures and evolutionary trends, with clustering results not directly equating to causal relationships or clinical evidence.

Ethics approval was not required because this bibliometric study analyzed published literature and did not involve human participants or identifiable private data.

## 3. Results

### 3.1. Frequency analysis of publication output

**Figure 2.**
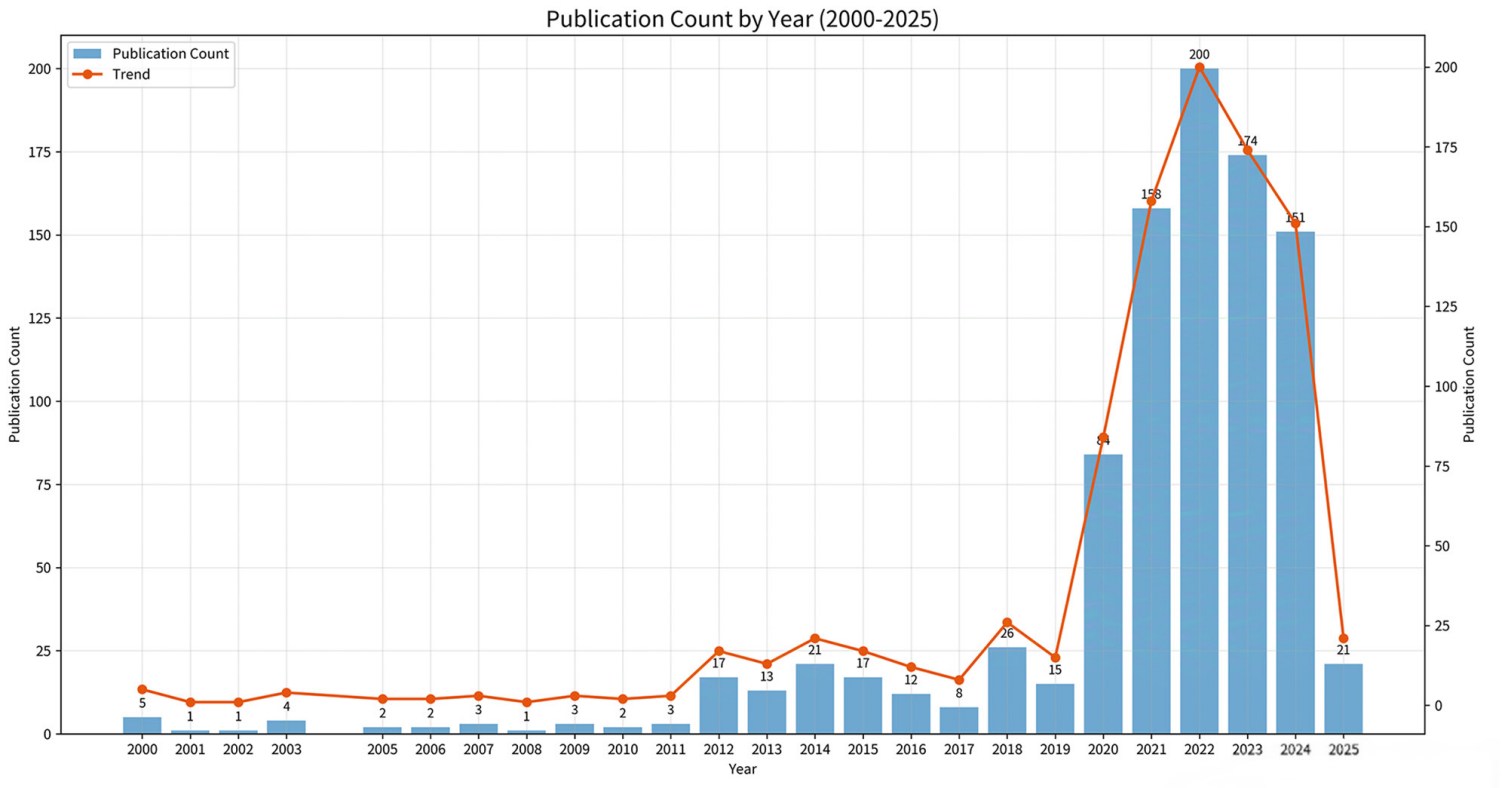
Annual publication volume from 2000 to 2025. The data for 2025 includes records published up to March 2025, as captured in the database on April 7, 2025.

Ultimately, this study included 944 valid publications spanning 378 academic journals, involving 6498 authors and 4657 research institutions. Annual publication trends are shown in Figure 2. Since 2020, the publication output in this field has shown a significant upward trend, peaking in 2022. An analysis combining retrieval processes, social context, and keywords revealed that the surge in publication output is associated with the COVID-19 outbreak, which has substantially accelerated research progress in this domain. This pattern suggests that public health emergencies may coincide with the accelerated research activities in this field.

### 3.2. Analysis of major institutions, countries, and authors

**Figure 3.**
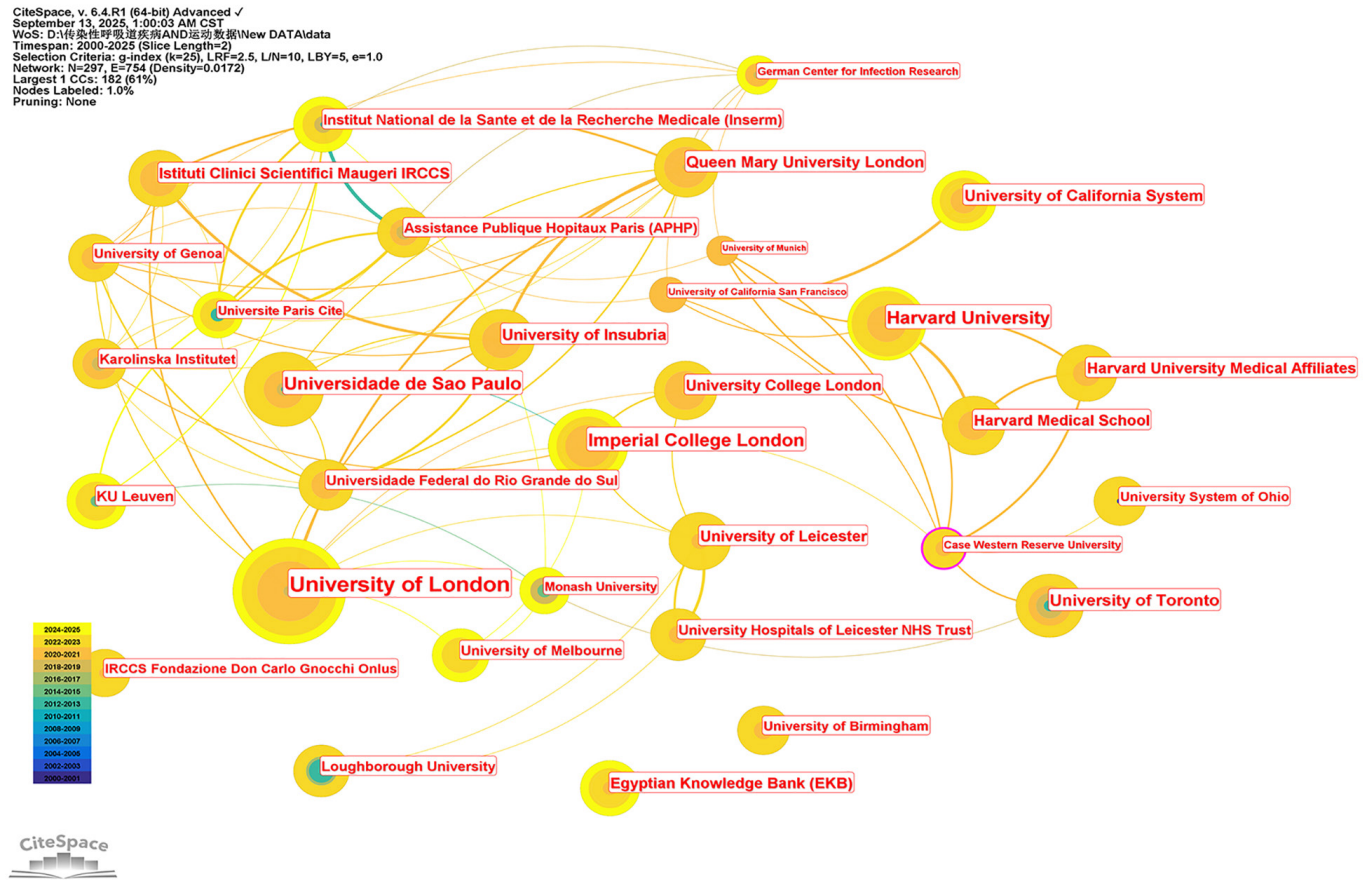
Research institution collaboration network map.

The collaborative network of the top 20 institutions by publication output is shown in Figure 3, and the top 10 institutions and authors are detailed in Table 2. The institutions with the highest publication output included the University of London (38 publications), followed by Harvard University, Universidade de São Paulo, and Imperial College London (all with 19 publications each). Research institutions from the United States and the United Kingdom have demonstrated substantial publication outputs. As indicated in Figure 3, Case Western Reserve University in the United States exhibits the strongest centrality among the institutions. The most prolific author is Arena Ross, who published multiple articles on exercise interventions for post-COVID-19 recovery and cardiorespiratory rehabilitation following infection. Other highly productive contributors include Visca Dina and Migliori Giovanni Battista.

**Table 2:** Top 10 institutions by post & authors by publication volume.

Institutions	Count	Authors	Count
University of London	38	Arena Ross	10
Harvard University	19	Visca Dina	8
Universidade de Sao Paulo	19	Migliori Giovanni Battista	8
Imperial College London	19	Singh Sally J	7
University of Toronto	14	Centis Rosella	7
University of California System	13	Gleeson Michael	6
University of Insubria	13	Borghi-silva Audrey	6
Queen Mary University London	13	Gleeson M	5
Harvard Medical School	12	Barton Andy	5
University College London	12	Jones Rupert	5

**Figure 4.**
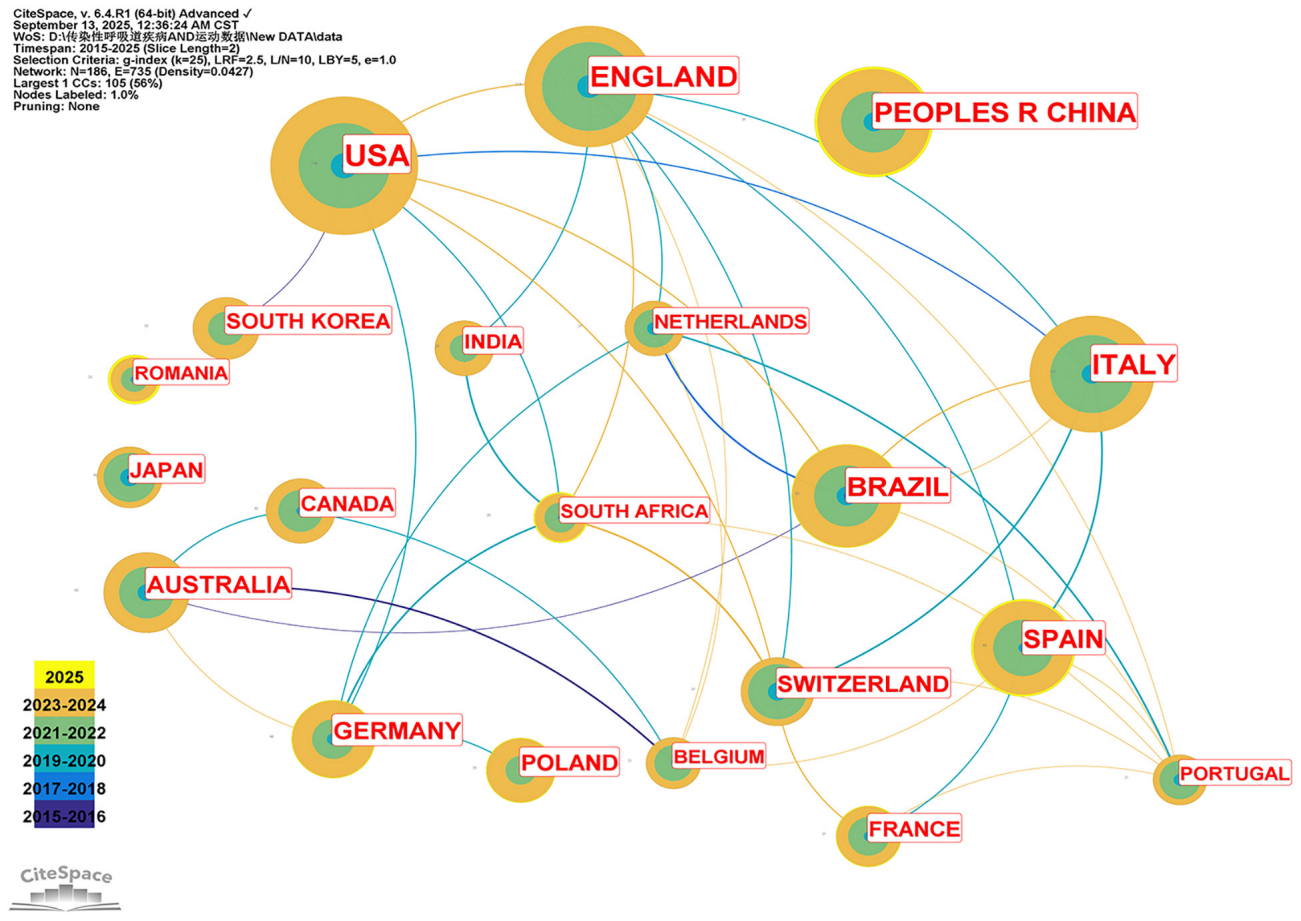
Country cooperation network map.

Figure 4 illustrates the collaborative publication network among the top 20 countries according to the publication volume. Countries with high publication output include the United States (161 publications), the United Kingdom (128 publications), China (115 publications, including Hong Kong, Macau, and Taiwan; Fig. 4 node labels reflect only 91 publications from Mainland China), Italy (107 publications), Brazil (87 publications), and Spain (71 publications). The earliest publication originated in Australia (2000), followed by the United States (2003). Although China ranks third in total publication volume, an analysis of per-author output reveals that Chinese authors have relatively lower individual publication counts, with contributions dispersed across many authors. Conversely, other leading countries have demonstrated robust academic collaborations, indicating sustained global research activity in this field.

### 3.3. Analysis of highly cited publications and journals

**Figure 5.**
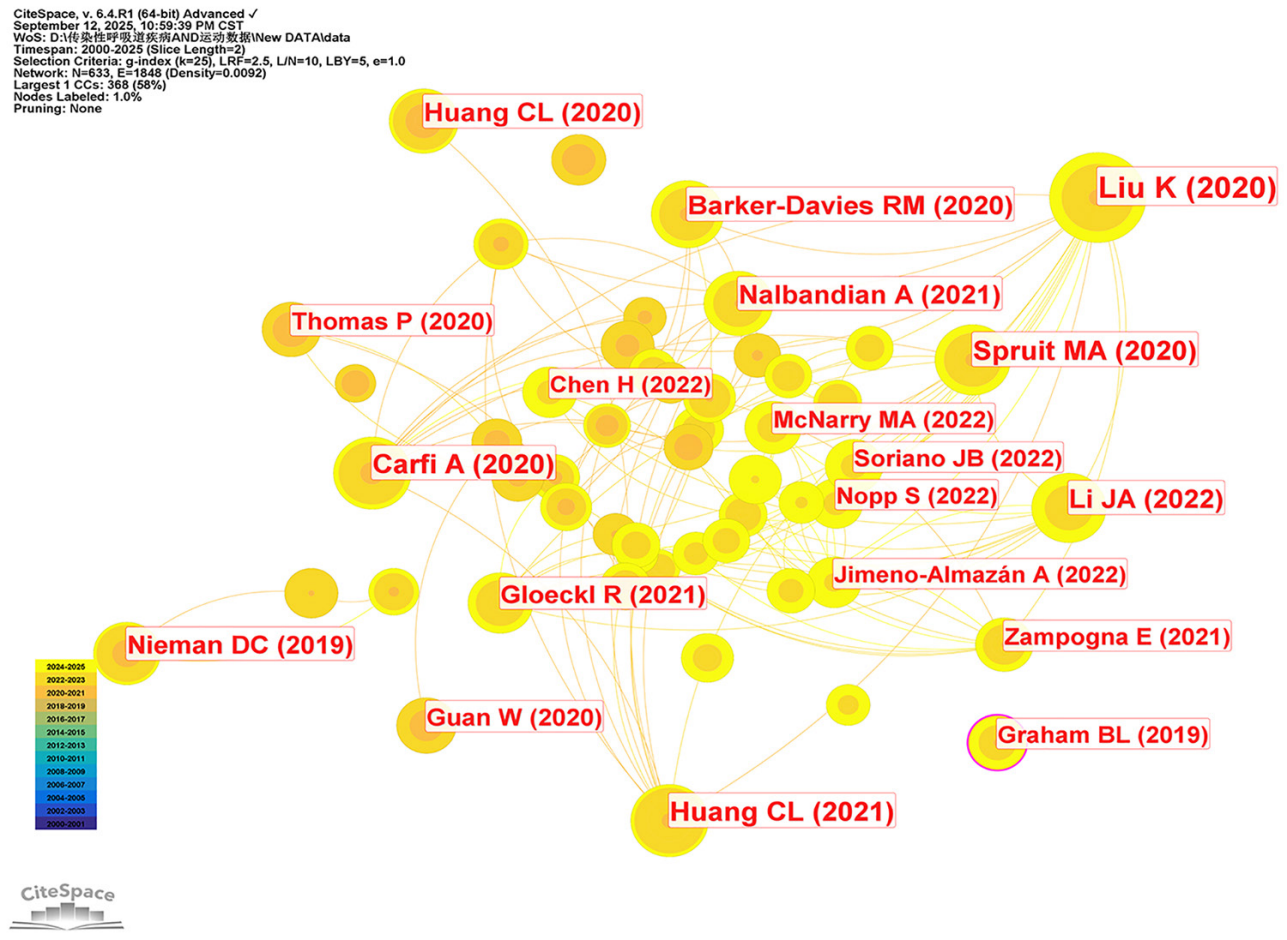
Literature co-citation network map.

**Figure 6.**
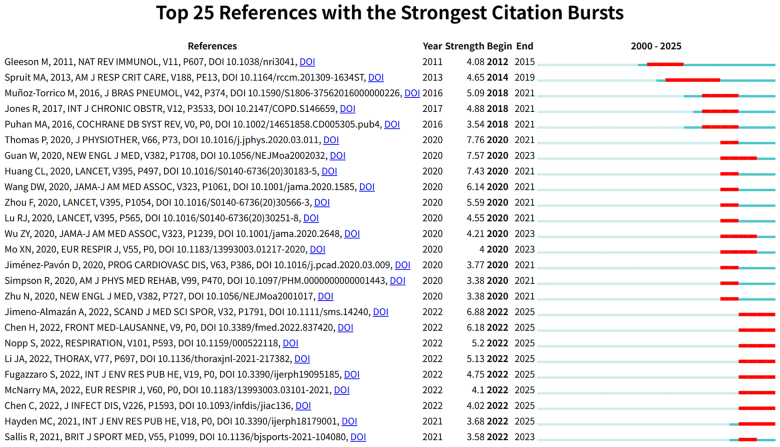
Top 25 cited references.

**Figure 7.**
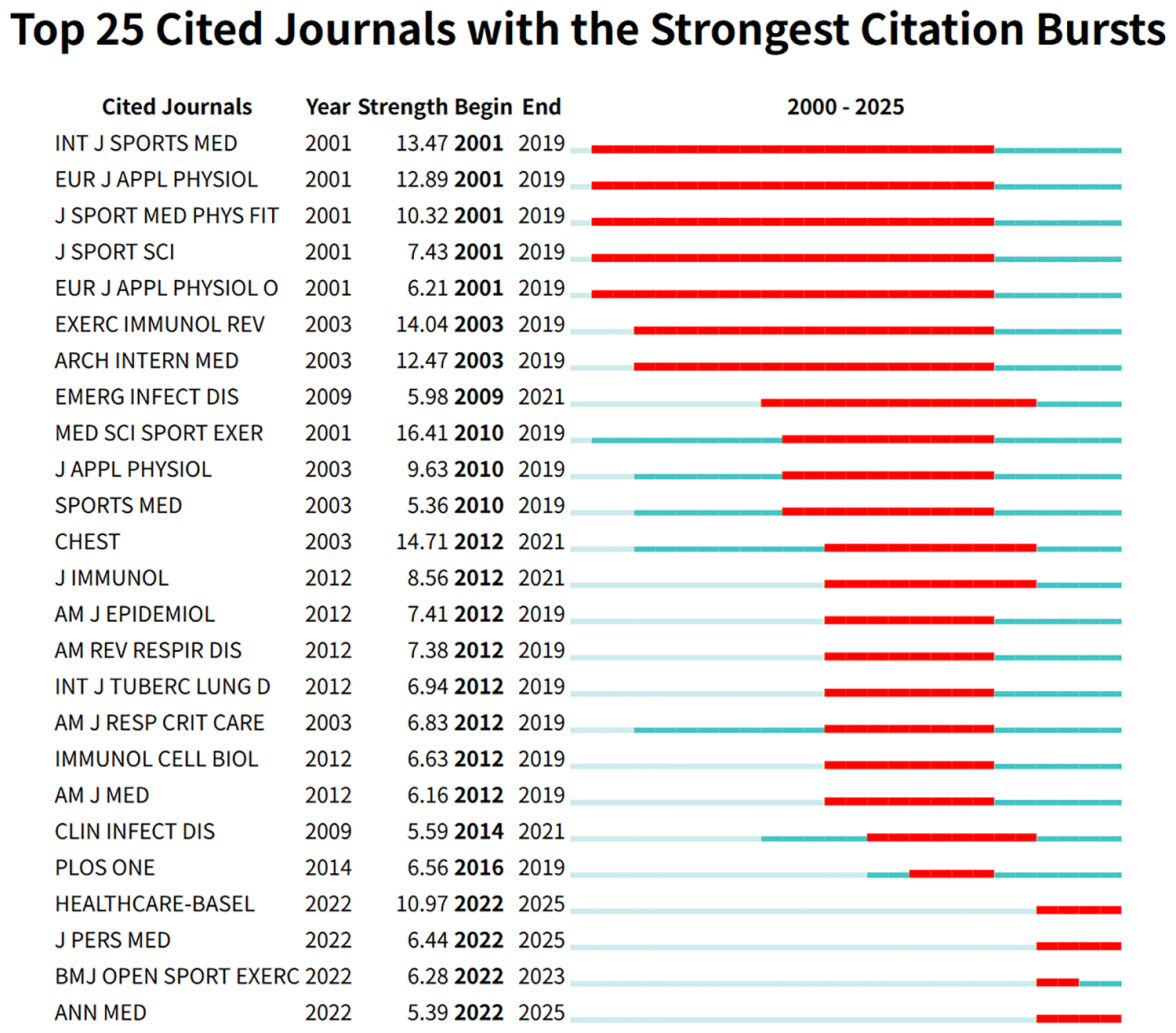
Top 25 cited journals.

The top 5 most-cited publications are listed in Table 3. The most-cited work, authored by Liu et al, was a randomized controlled trial (RCT) on respiratory rehabilitation for elderly COVID-19 patients,^[19]^ accumulating 91 citations. In a study on COVID-19 patients 6 months post-discharge, Huang ranked second in citation frequency with 59 citations.^[20]^ This was followed by Carfi A, whose research focused on persistent symptoms within 60 days post-COVID-19 infection.^[21]^ These studies reported fatigue and dyspnea as common persistent symptoms among COVID‑19 survivors, which have been frequently discussed in relation to rehabilitation and exercise‑based interventions. Figure 5 presents the co-citation network of references, with the highest citation burst centrality attributed to Graham’s 2019 spirometry standardization update (Graham, 2019, AM J RESPIR CRIT CARE, V200, PE70, digital object identifier 10.1164/rccm.201908-1590ST) published in Am J Respir Crit Care Med. Figures 6 and 7 show the top 25 burst-strength cited references and co-cited journals from January 2000 to March 2025. The red segments indicate the duration of the citation bursts.

**Table 3:** Top 5 cited publications.

Count	Centrality	Year	Cited references
91	0.08	2020	Liu K. 2020. Complement Ther Clin, V39, P0, DOI 10.1016/j.ctcp.2020.101166
59	0.03	2021	Huang CL. 2021. Lancet, V397, P220, DOI 10.1016/S0140-6736(20)32656-8
58	0.03	2020	Carfi A. 2020. JAMA-J Am Med Assoc, V324, P603, DOI 10.1001/jama.2020.12603
56	0.01	2020	Spruit MA. 2020. Eur Respir J, V56, P0, DOI 10.1183/13993003.02197-2020
53	0.09	2020	Barker-Davies RM. 2020. Brit J Sport Med, V54, P949, DOI 10.1136/bjsports-2020-102596

The original 2021 Lancet article was later retracted and republished in 2023 (Lancet 401(10393): e21–e33; DOI: 10.1016/S0140-6736(23)00810-3). Bibliometric indicators were computed based on records indexed in WOSCC at the time of data extraction.

DOI = digital object identifier, P = page, V = volume.

### 3.4. Keyword analysis

Keyword analysis can reveal the research directions and short-term trends in this field. Given that publication volume has been concentrated over the past decade, and to enhance the focus of the analysis, this study conducted keyword analysis on a decade-long sample. Using the g-index (*k* = 10) for filtering, 331 keyword nodes were obtained for the subsequent analysis.

#### 3.4.1. Keyword co-occurrence analysis

**Figure 8.**
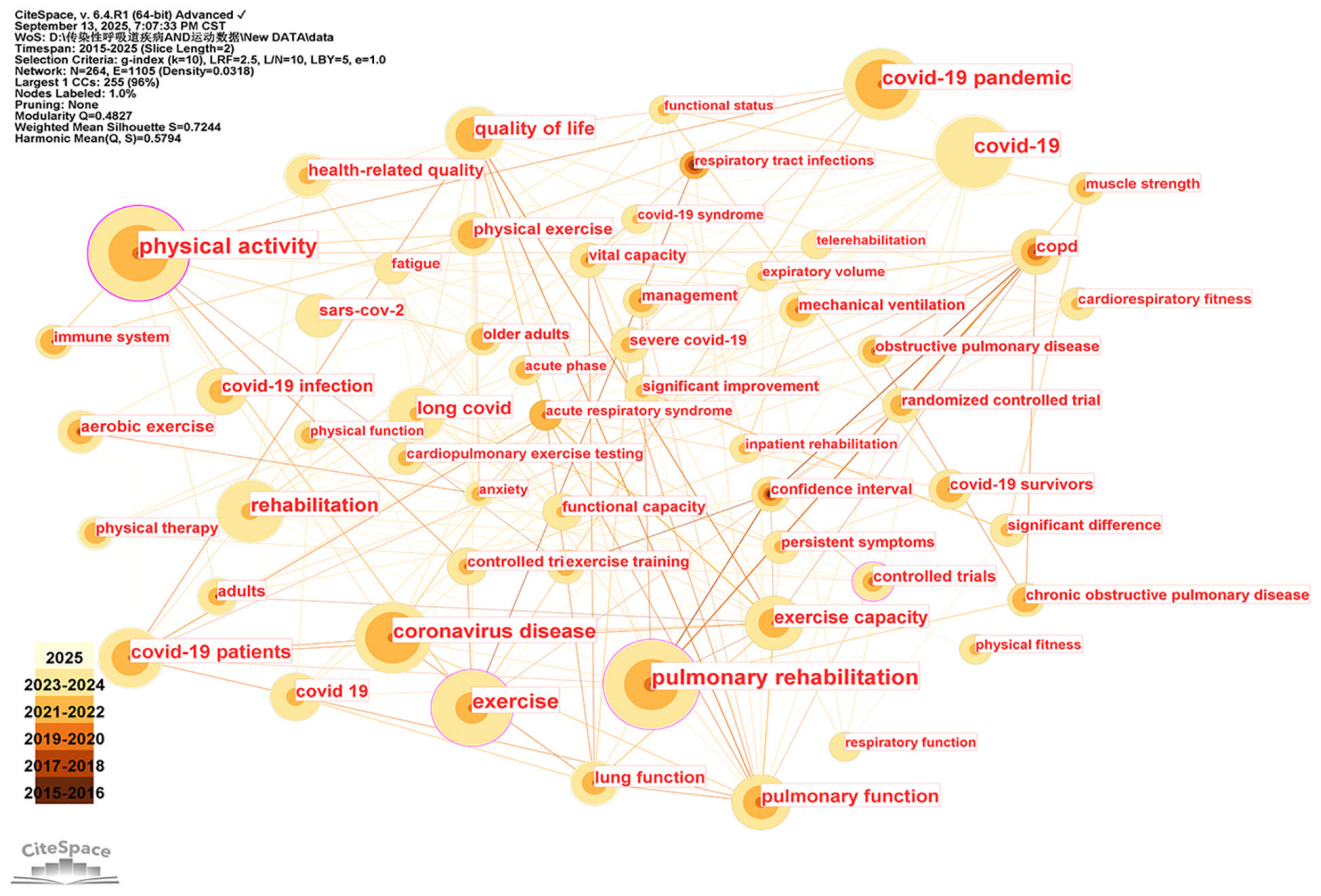
Co-occurrence map of keywords.

Keywords with an occurrence frequency of ≥20 were screened, yielding the keyword co-occurrence map shown in Figure 8. High-frequency keywords included “Physical activity” (226 occurrences), “Pulmonary rehabilitation” (193 occurrences), “Exercise” (145 occurrences), “COVID-19” (137 occurrences), “Coronavirus disease” (125 occurrences), “Rehabilitation” (102 occurrences), and “Quality of life” (80 occurrences). These keywords exhibited a high frequency of occurrence within the network, with “physical activity,” “exercise,” and “pulmonary rehabilitation” demonstrating high centrality within the co-occurrence network.

#### 3.4.2. Keyword cluster analysis

**Figure 9.**
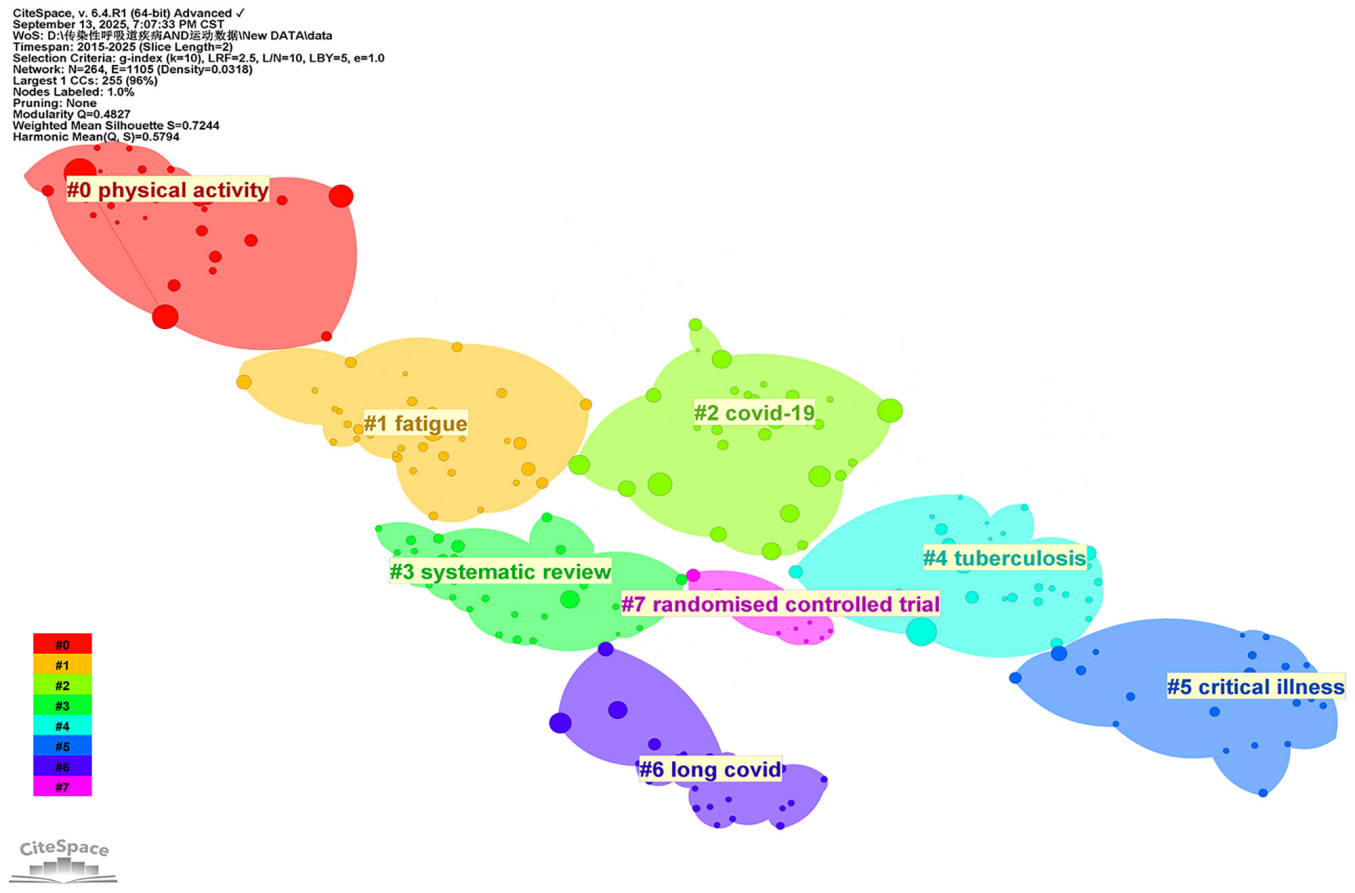
Keyword clusters were identified by utilizing the LLR statistic. LLR = log-likelihood ratio.

Using CiteSpace, we performed keyword cluster analysis and identified 8 cluster labels through log-likelihood ratio clustering based on similarities in research keywords, topics, or literature: “Physical activity,” “Fatigue,” “COVID-19,” “Systematic review,” “Tuberculosis,” “Critical illness,” “Long COVID,” and “Randomised controlled trial.” As shown in Figure 9, both the modularity value (*Q* = 0.487 > 0.3) and average contour coefficient (*S* = 0.7244 > 0.5) meet the threshold criteria, indicating that the clustering structure possesses acceptable distinctiveness and consistency.

The analysis of keywords maps a distinct evolution in the research paradigm for exercise interventions in respiratory infections over the past decade, demarcated by the COVID-19 pandemic. A stable core, defined by “physical activity,” “exercise,” and “pulmonary rehabilitation,” consistently targets functional and quality-of-life outcomes. The pandemic then triggered a paradigm shift, rapidly moving the focus from “Tuberculosis” to “COVID-19,” “Critical illness” and subsequently to the “Long COVID” cluster with its associated “fatigue” and “dyspnea.” The current frontier, revealed by strong burst keywords like “Telerehabilitation” and “Respiratory function,” is characterized by the increasing attention to digitally delivered rehabilitation modalities and related outcome reporting.

#### 3.4.3. Keyword hotspot analysis and burst detection

**Figure 10.**
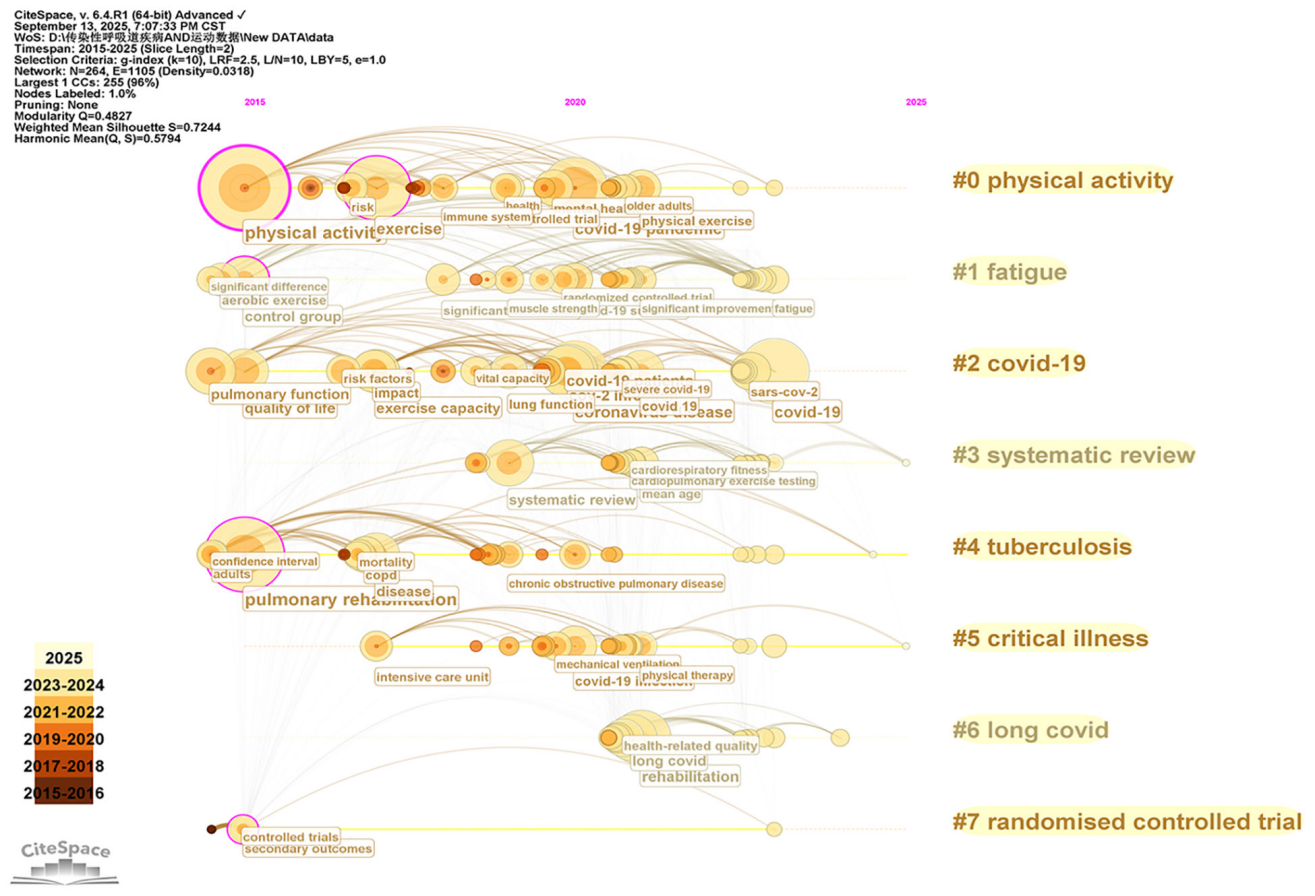
Dynamic keyword evolution map.

**Figure 11.**
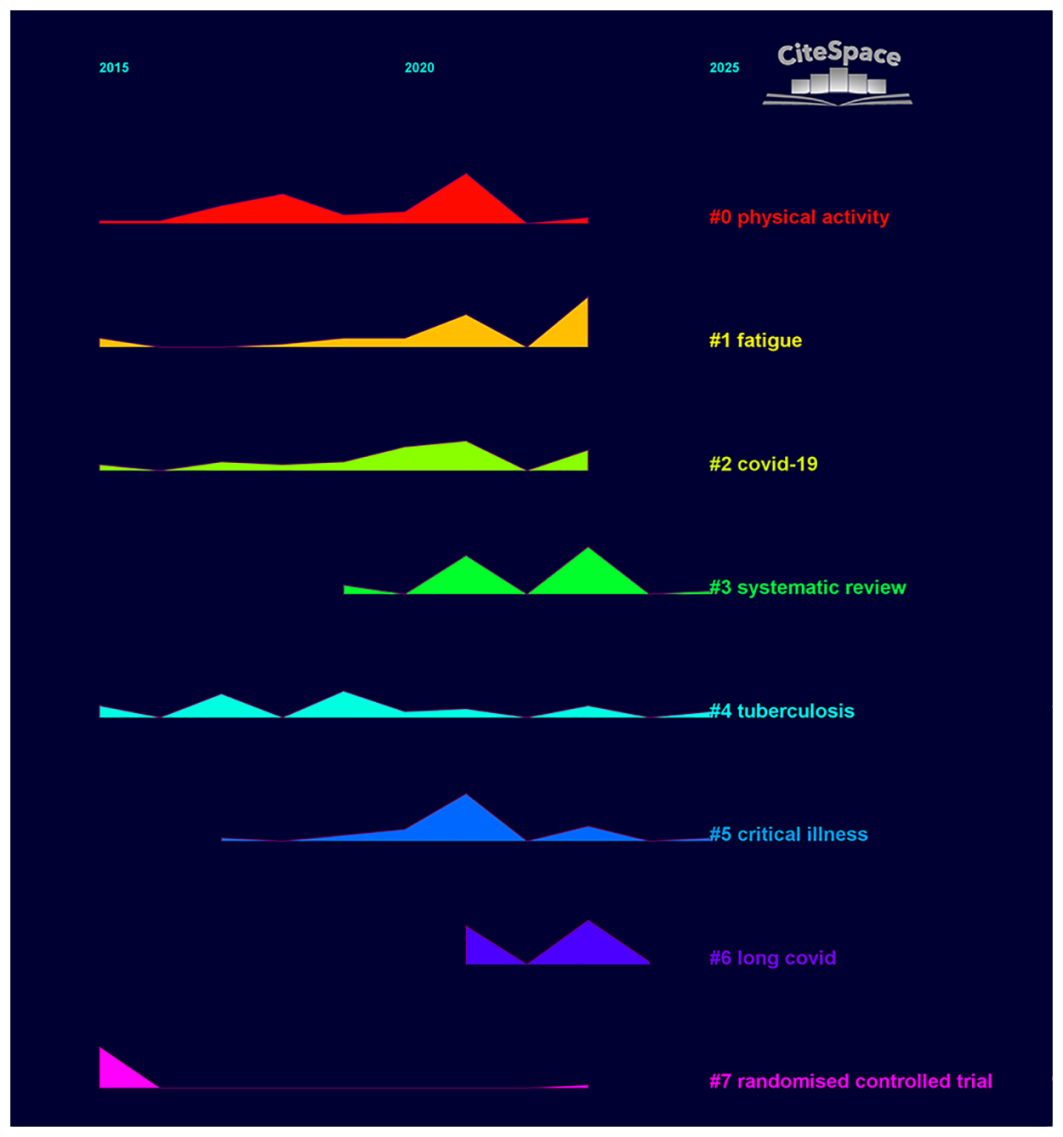
Dynamic research cluster theme trends map.

Figure 10 illustrates the temporal evolution of keywords across different cluster-labeled research domains within this field over the past decade. Figure 11 highlights the trends and popularity distribution of various cluster labels in this research area during the last 10-year period. From Figures 10 and 11, we observe that all clusters except #4 “Tuberculosis” and #7 “Randomised controlled trial” exhibit peak keyword popularity concentrated between 2019 and 2022. Only cluster #4 “Tuberculosis” shows a primary keyword concentration predating 2019, further indicating that research in this field has been predominantly driven by the COVID-19 outbreak. However, the diagram exhibits certain clustering errors, as keywords clustered under “COVID-19” appear distributed during the 2015 to 2019 period. No research related to COVID-19 has been conducted prior to 2019. Through research group discussions and synthesis, we identified that some pre-2019 keywords were associated with atypical coronavirus infection. Consequently, the analytical software classified these keywords using the COVID-19 cluster labels. Nevertheless, the primary research trend peak emerged after 2019, thereby confirming the rationality of the research trajectory.

**Figure 12.**
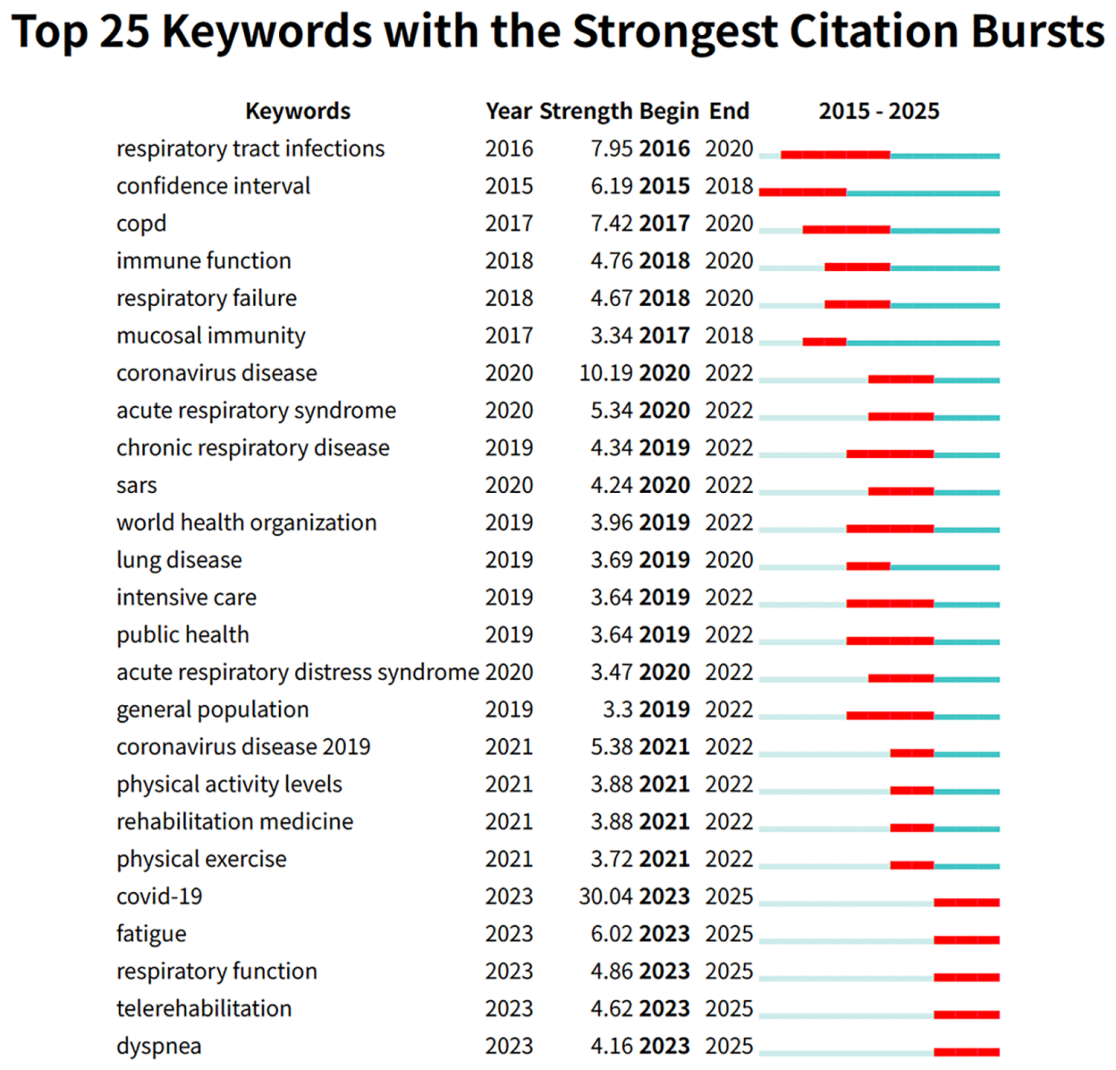
Bursting keywords.

Figure 12 displays the keyword burst detection, revealing research hotspots, their centrality strength, and the top 25 burst keywords. This illustrates the emergence of research hotspots at different times in the past decade. Keywords exhibiting strong burst strength include “COVID-19” (30.04), “Coronavirus disease” (10.19), and “respiratory tract infections” (7.95). Recent high-burst keywords encompass “COVID-19” (30.04), “Fatigue” (6.02), “Respiratory function” (4.86), “Telerehabilitation” (4.62), and “dyspnea” (4.16). The evolution of burst keywords reveals that the research focus in this field has progressively shifted from early investigations into the immune mechanisms of respiratory infections toward pulmonary function and critical rehabilitation in COVID-19 patients, further expanding into telerehabilitation modalities.

## 4. Discussion

With population mobility, climate variability, and global aging.^[22]^ Against this backdrop, to advance academic research on exercise interventions for respiratory infectious diseases, the findings of this study can help researchers in the field quickly grasp the current research hotspots, knowledge structure, and emerging priorities. This bibliometric study maps the research landscape in this field over the past 25 years, and we will discuss it in terms of its developmental stages, thematic frameworks, and cutting-edge directions.

### 4.1. Overall trends: a research field shaped by public health crises

Based on an analysis of the research findings, the evolution of this field follows a clear trajectory, shifting from stable, disease-specific foundational work to a rapid, event-driven paradigm shift. Based on the burst keyword “COVID-19” and the research trend showing a surge in publication volume beginning in 2019 to 2020, it can be broadly inferred that this shift was catalyzed by the COVID-19 pandemic. In conjunction with the temporal structure shown in Figures 10–12, the research landscape can be divided into 3 phases to explain the dramatic changes in research focus and scale.

#### 4.1.1. Foundational period (pre-2020)

There is relatively little literature available prior to 2020. By combining the keyword trends shown in Figure 10, the changes in clustering module trends depicted in Figure 11, and the emerging burst keywords identified in Figure 12 for this period, it can be broadly inferred that research during this phase focused more on chronic respiratory sequelae and established exercise intervention scenarios.^[23,24]^

#### 4.1.2. Catalytic crisis and paradigm formation phase (2020–2023)

Similarly, based on the changes in publication trends shown in Figure 2 and the keyword patterns in Figures 10–12, the field has expanded significantly since 2020. It can be inferred that the COVID-19 pandemic had a profound catalytic effect on research in this field, triggering a significant increase in the number of related publications. Notably, as shown by the emerging keywords in Figure 12, “telerehabilitation” began to emerge in 2023, indicating that the field of telerehabilitation intervention research has come into sharper focus. The social model of home isolation during the COVID-19 pandemic propelled a previously niche form of exercise intervention “telerehabilitation” into the spotlight of research in this field.

### 4.2. Paradigm evolution of research foci: from a stable foundation to a technologically empowered frontier

Examining the evolution of keyword clusters and timelines (Figs. 9–11) shows that research themes have been substantially restructured over time; additionally, Figure 12, using keyword burst analysis, illustrates how keyword hotspots shifted across periods.

Early research primarily focused on themes such as physical activity, exercise, and pulmonary rehabilitation, and on functional recovery and quality-of-life outcomes. Previous studies have reported associations between exercise-related factors and immune and inflammatory markers.^[9,12,25,26]^ These findings align with the burst detection analysis in Figure 12, where “immune function” exhibited a high burst intensity during the early phase of research. This may explain why certain research themes remained relatively stable during the early period.^[26–28]^ Prior to the pandemic, research predominantly centered on lung damage associated with lower respiratory tract infections (such as post-TB pulmonary disease),^[28,29]^ and the prevention and symptomatic management of upper respiratory tract infections.^[5,30]^ With the outbreak of the COVID-19 pandemic, the research agenda in this field has undergone a significant shift. The focus has rapidly transitioned from chronic sequelae such as “tuberculosis” to the acute management of COVID-19, “critical illness,” and “long COVID,” whose primary clinical features include “fatigue” and “dyspnea.” During the pandemic, attention was expanded to include studies on the role of exercise in lung function and symptom management in patients with acute and convalescent respiratory infections.^[31]^

Against the backdrop of this shift in research focus, intervention models have evolved. Key terms such as “telerehabilitation” and “respiratory function” have gained prominence, signaling a trend toward integrating digital health technologies with traditional rehabilitation approaches.^[32–35]^

### 4.3. Paradigm shift in intervention models and evolution of rehabilitation frameworks

The clustering theme evolution trends and burst keyword analysis are shown in Figures 11 and 12. Based on trends over time, we can generally infer that the research framework for this topic has evolved in response to changes over time and shifts in the societal burden of disease.

Based on the results of the visual network analysis, research prior to the COVID-19 pandemic focused primarily on 2 areas: long-term management of post-TB lung disease; and the role of exercise in the prevention of upper respiratory tract infections.^[10]^ The pandemic triggered a significant reconfiguration, as illustrated in Figures 10 and 11 (Cluster #5), with a surge in publications between 2020 and 2022, where the research focus rapidly shifted toward studies discussing early exercise interventions during the acute phase of critical infections, including feasibility- and implementation-related themes. Notably, the COVID-19 rehabilitation guidelines proposed by Spruit et al have been frequently cited and discussed in the literature.^[36]^ This guideline systematically proposes a 3-stage framework of “acute phase management – subacute rehabilitation – long-term sequelae intervention,” which has been frequently cited and discussed in subsequent literature. These co-cited guidelines converge on a staged rehabilitation framework that is frequently discussed in the literature. Post‑TB lung disease may represent a relevant context in which the transferability of such staged approaches could be empirically evaluated, given recent qualitative work on rehabilitation needs and programmatic feasibility studies of post‑TB assessment pathways.^[37,38]^ Traditionally, TB rehabilitation typically commences after anti-infection treatment. Future studies could evaluate whether such staged rehabilitation frameworks can be adapted to TB and other respiratory infections, including whether earlier integration of exercise/rehabilitation during anti‑infective treatment is feasible, safe, and effective.

### 4.4. Core controversies in exercise interventions for respiratory tract infections

Based on current research, exercise rehabilitation remains a highly controversial and limited intervention for respiratory infectious diseases. A primary area of uncertainty involves the optimal exercise intensity. Although some studies have identified a “J-shaped curve” relationship between exercise intensity and infection risk,^[39]^ specific intensity thresholds and dose-response effects for distinct populations remain unestablished.^[10,29,36,40]^ Furthermore, the precise mechanism of action is still debated. It is unclear whether the associated increased risk arises from physiological immune modulation following exercise or from environmental exposure factors.^[41,42]^ Finally, intervention strategies themselves lack clarity, with insufficient definitive evidence concerning the necessity of exercise during upper respiratory tract infections, the safest timing, appropriate types and intensities,^[43,44]^ as well as the net effects on symptom outcomes, quality of life, and disease progression.^[45,46]^

### 4.5. Future trends and prospects of exercise interventions in respiratory infectious diseases

Analysis of research hotspot trends in Figure 12 indicates increasing research attention for telerehabilitation. Publication attention to telerehabilitation rose sharply during COVID‑19. While an RCT in long COVID reported favorable outcomes,^[32]^ the wider evidence base remains under synthesis,^[33]^ and bibliometric mapping cannot be used to infer intervention effectiveness.

### 4.6. Limitations and deficiencies

This study had several limitations. First, the data sources were limited to English literature from the WOSCC and PubMed databases, excluding research outputs in other languages. Consequently, research data from non-English-speaking countries may not be fully captured.

Our search strategy also has its limitations. The initial yields differed substantially between WOSCC and PubMed, reflecting their distinct indexing scopes. However, all bibliometric analyses were conducted on a merged and de-duplicated dataset after applying uniform screening criteria. This process mitigates potential bias from the coverage of any single database, and our interpretations focus on macro-level patterns derived from this synthesized corpus.

Second, during the screening of a large volume of literature, we relied solely on the inclusion and exclusion criteria (such as whether the study’s content aligned with the topic) without conducting individual assessments of each study’s quality, methodological rigor, or the strength of evidence supporting its conclusions. In addition, we did not stratify the included records by study design (e.g., randomized trials, observational studies, and reviews), which limits the interpretation of the evidence composition behind each theme.

Third, the research trends revealed through bibliometric analysis largely depend on software algorithms and are subject to identification bias. For instance, the #2 “COVID-19” cluster shown in Figures 10 and 11 emerged during 2015 to 2019. However, no actual “COVID-19” related research existed during this period. Through multiple renderings and cross-verification with raw data, we concluded that this error likely stemmed from software misclassification of pre-pandemic exercise studies on atypical coronavirus pneumonia under the COVID-19 theme. Despite such deviations, the overall temporal patterns remain informative for describing publication dynamics and thematic structures.

Because bibliometric networks are sensitive to parameter choices, cluster structures and node-level metrics – such as betweenness centrality and burst detection – may shift when settings like node-selection thresholds or time-slicing schemes are adjusted. In this study, we interpreted clusters as heuristic thematic groupings and treated centrality/burst results as exploratory indicators rather than parameter-invariant evidence. Our main conclusions rely on convergent macro-level patterns (publication growth, recurring thematic cores, and dominant hotspot directions) rather than any single clustering solution or single-node metric. In the keyword analysis, this study employed g-index *K*-values that differed from default settings. To mitigate potential data bias, the results were subjected to sensitivity analyses using different values of *K (k* = 10, 15, and 25). The results indicate that, although there were slight and expected fluctuations in network structure and node centrality rankings due to the different *K* values, the core high-frequency keywords and the structure of the major thematic clusters remained relatively stable, with no impact on the overall findings. Therefore, we regard the results derived from the centrality and burstiness analyses as exploratory rather than definitive evidence. Our main conclusions are based on macro-level patterns such as publication growth trends, recurrent thematic cores, and dominant cluster topics, which remain stable across different parameter settings.

In summary, constrained by the aforementioned factors, this study offers only limited insights for recent research in this field and cannot accurately predict macro-level and long-term research directions. Readers should interpret these findings in context, as bibliometric results primarily reflect publication patterns and thematic emphasis rather than evidence quality or clinical recommendations.

## 5. Conclusions

Over the past 25 years, research on exercise interventions for respiratory infectious diseases has shown sustained growth, with explosive expansion occurring during the COVID-19 pandemic. Results from bibliometric and visualization analyses indicate that research hotspots in this field have long centered on themes such as physical activity, exercise, and pulmonary rehabilitation. In recent years, there has been a marked shift toward COVID-19-related studies as the core focus, accompanied by the persistent emergence of themes including “fatigue,” “respiratory function,” “dyspnea,” and “telerehabilitation.”

This study systematically delineates the knowledge structure, distribution of research themes, and temporal evolution characteristics within this field from a bibliometric perspective, thus providing a macro-level perspective for understanding shifts in research agendas across different phases. It is important to emphasize that these findings primarily reflect shifts in research focus and thematic evolution and do not constitute direct evidence regarding the clinical efficacy or comparative merits of specific exercise intervention programs.

## Author contributions

**Conceptualization:** Qiugang Zheng, Jindong Chen, Na Wang.

**Data curation:** Qiugang Zheng, Jindong Chen, Na Wang.

**Formal analysis:** Qiugang Zheng, Jindong Chen, Na Wang.

**Funding acquisition:** Qiugang Zheng, Zan Liu.

**Investigation:** Qiugang Zheng, Jindong Chen, Na Wang.

**Methodology:** Qiugang Zheng, Jindong Chen, Na Wang.

**Project administration:** Qiugang Zheng, Jindong Chen, Na Wang.

**Resources:** Qiugang Zheng, Jindong Chen, Na Wang.

**Software:** Qiugang Zheng, Jindong Chen, Na Wang.

**Validation:** Qiugang Zheng, Jindong Chen, Na Wang.

**Visualization:** Qiugang Zheng, Jindong Chen, Na Wang.

**Supervision:** Xiurong Xu.

**Writing – original draft:** Qiugang Zheng, Na Wang.

**Writing – review & editing:** Na Wang, Mengnan Hou, Xiurong Xu, Zan Liu.
